# Supplementary material for: Modeling In Vitro Osteoarthritis Phenotypes in a Vascularized Bone Model Based on a Bone-Marrow Derived Mesenchymal Cell Line and Endothelial Cells
Source: Int J Mol Sci. 2021 Sep 3;22(17):9581. doi: 10.3390/ijms22179581 (PMC8430538; doi:10.3390/ijms22179581)
Supplement: Supplementary file 1 [file ijms-22-09581-s001.zip › ijms-1303860-supplementary.pdf]

Supplementary material for the manuscript: **Modeling in vitro osteoarthritis phenotypes in a vascularized bone model based on a bone-marrow derived mesenchymal cell line and endothelial cells**

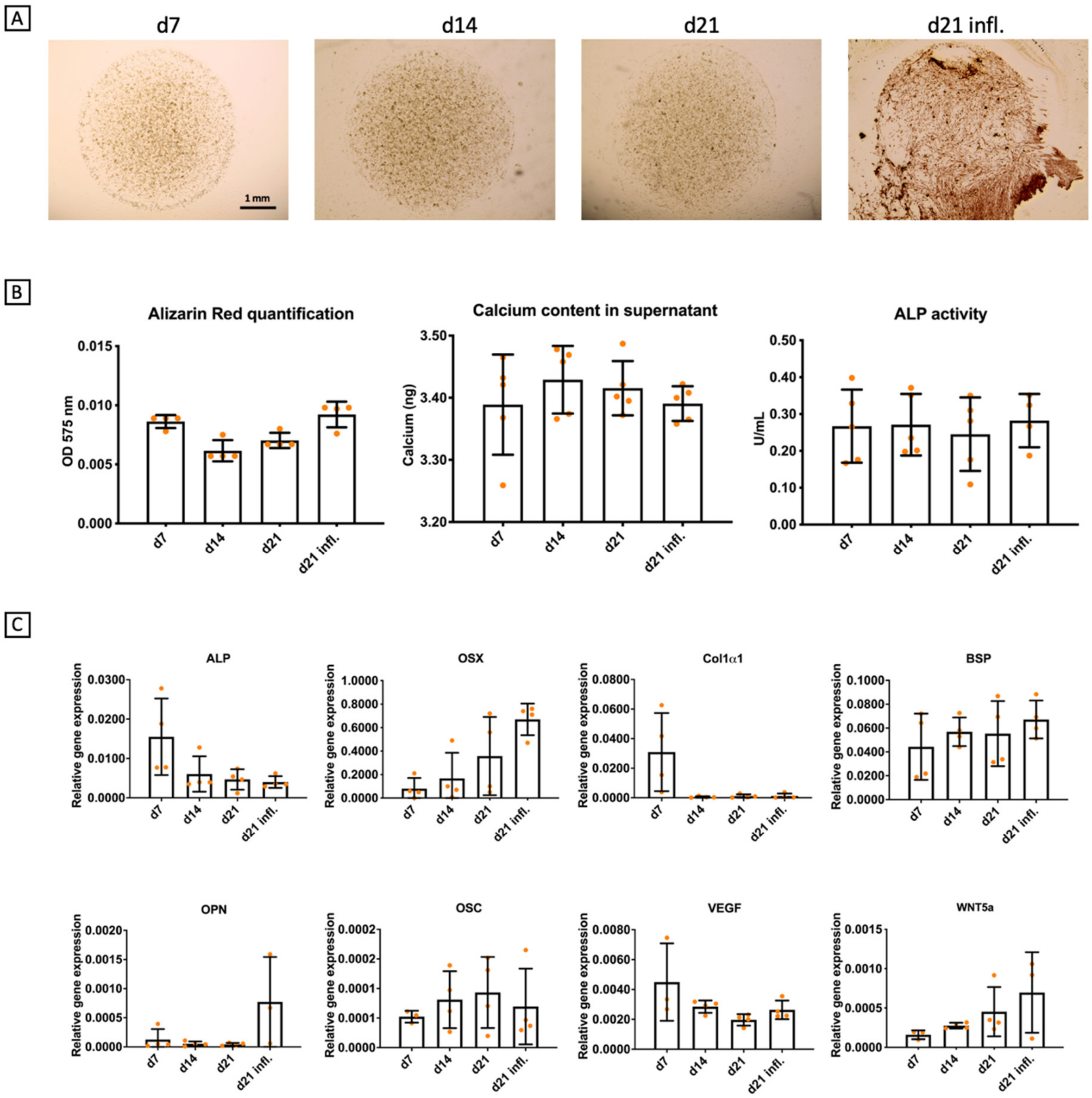

Figure S1: Osteogenic characterizations of HUVECs group. (**A**) Alizarin red staining of HUVECs during culture. No calcium accumulation was detectable in the matrix, except for a slight increase after inflammation which resulted however to be below the detection limit during quantifications, and not comparable to the amounts measured in the MSOD or MSOD-HUVEC groups; (**B**) Supernatant analyses showing no calcium accumulation reported in terms of alizarin red staining, calcium released in the medium, and ALP activity levels which in all cases were not comparable to the values of MSOD and MSOD-HUVEC groups; (**C**) Quantitative RT-PCR analyses of osteogenic genes. Values were not comparable to the ones detected in MSOD and MSOD-HUVEC groups. No statistical differences were registered due to culture time or inflammation. ALP = alkaline phosphatase, COL1 = collagen type I, OSC = osteocalcin, VEGF = vascular endothelial growth factor, MMP13 = matrix metallopeptidase 13, ADAMTS5 = A disintegrin and metalloproteinase with thrombospondin motifs 5. Gene expression is relative to GAPDH levels. Number of experiments = 3, number of replicates/experiment = 5. ns = non-significant, * = $p<0.05$, ** = $p<0.005$, *** = $p<0.0005$, **** $p<0.00005$.

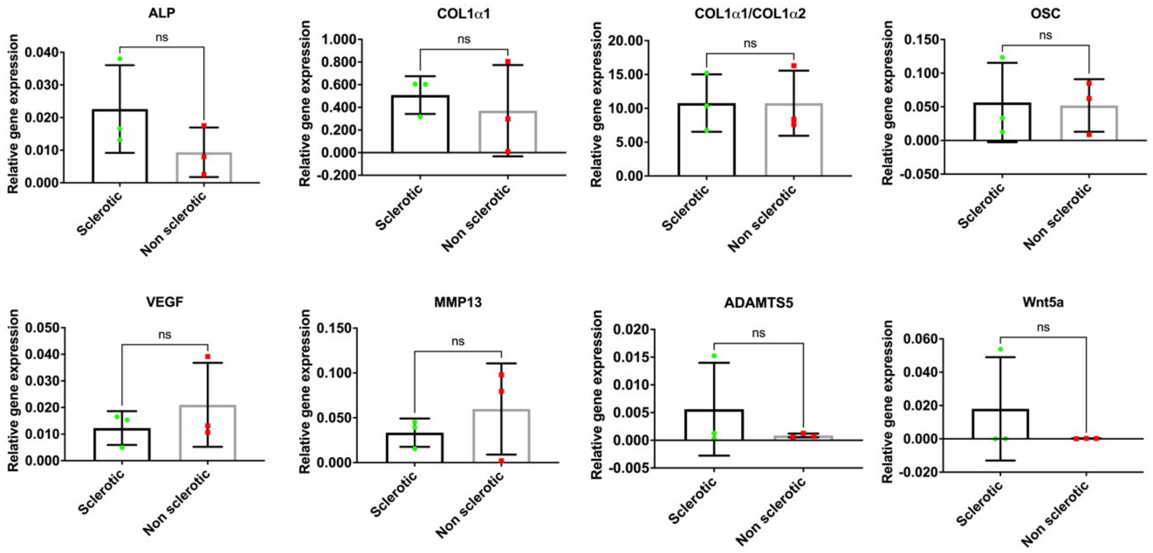

Figure S2: Quantitative RT-PCR analysis of human OA bone. Sclerotic vs non-sclerotic bone pieces were isolated from the tibial plateau of OA patients who underwent total knee angioplasty. No statistically significant differences were observed between sclerotic and non-sclerotic bone in the analyzed genes. Number of donors = 3, number of replicates = 3. ns = non-significant. ALP = alkaline phosphatase, COL1 = collagen type I, OSC = osteocalcin, VEGF = vascular endothelial growth factor, MMP13 = matrix metallopeptidase 13, ADAMTS5 = A disintegrin and metalloproteinase with thrombospondin motifs 5. Gene expression is relative to GAPDH levels. ns = non-significant, * = p<0.05, ** = p<0.005, *** = p<0.0005, **** p<0.00005.

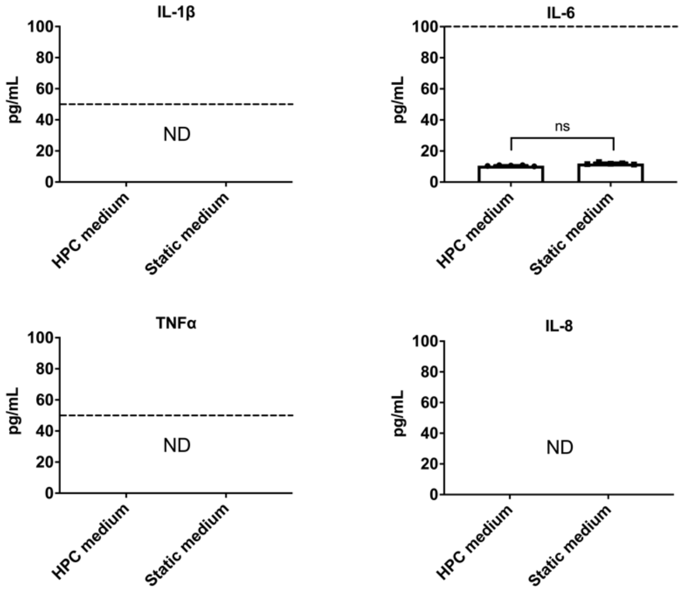

Figure S3: Inflammatory cytokines analysis of OA cartilage-on-chip conditioned medium. Different conditioned media were analyzed. A Luminex assay was used to check for IL-1β, IL-6 and TNFα concentrations; ELISA was used to check for IL-8 presence. Dashed lines represent the concentration of the cytokines used in the inflammation experiment on vascularized bone model. ns = non-significant, * = p<0.05, ** = p<0.005, *** = p<0.0005, **** p<0.00005.
